# 1*H*-Imidazo[4,5-*f*][1,10]phenanthrolin-7-ium perchlorate monohydrate

**DOI:** 10.1107/S1600536809034576

**Published:** 2009-09-05

**Authors:** Su-Mei Shen

**Affiliations:** a Department of Applied Engineering, Zhejiang Economic and Trade Polytechnic, 310018 Hangzhou, People’s Republic of China

## Abstract

In the title crystal structure, C_13_H_9_N_4_^+^·ClO_4_^−^·H_2_O, cations, anions and water mol­ecules are linked through inter­molecular N—H⋯O, O—H⋯N and O—H⋯O hydrogen bonds, forming layers parallel to (001). In addition, there are weak π–π stacking inter­actions between the layers, involving the cations and with centroid–centroid distances in the range 3.584 (2)–3.662 (2) Å, forming a three-dimensional network.

## Related literature

For background to 1*H*-imidazo[4,5-*f*][1,10]-phenanthroline and its use as a mol­ecular building block, see: Xiong *et al.* (1999 ▶); Yu *et al.* (2009 ▶); Liu *et al.* (2009 ▶).
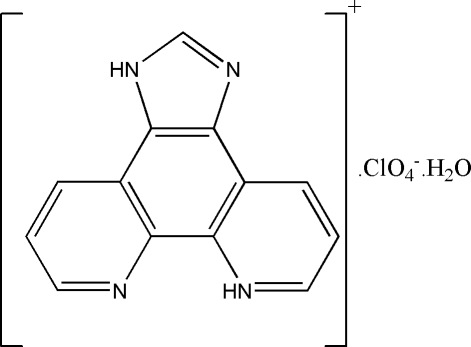


## Experimental

### 

#### Crystal data

C_13_H_9_N_4_^+^·ClO_4_^−^·H_2_O
*M* *_r_* = 338.71
Monoclinic, 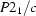

*a* = 11.401 (2) Å
*b* = 18.475 (3) Å
*c* = 6.7163 (13) Å
β = 90.179 (3)°
*V* = 1414.7 (4) Å^3^
*Z* = 4
Mo *K*α radiation
μ = 0.30 mm^−1^
*T* = 298 K
0.30 × 0.26 × 0.17 mm

#### Data collection

Bruker APEXII area-detector diffractometer
Absorption correction: multi-scan (*SADABS*; Bruker, 2005 ▶) *T*_min_ = 0.914, *T*_max_ = 0.950
7051 measured reflections
2534 independent reflections
1734 reflections with *I* > 2σ(*I*)
*R*_int_ = 0.031

#### Refinement

*R*[*F* ^2^ > 2σ(*F* ^2^)] = 0.057
*wR*(*F* ^2^) = 0.197
*S* = 1.01
2534 reflections
208 parameters
3 restraints
H-atom parameters constrained
Δρ_max_ = 0.41 e Å^−3^
Δρ_min_ = −0.39 e Å^−3^

### 

Data collection: *APEX2* (Bruker, 2005 ▶); cell refinement: *SAINT* (Bruker, 2005 ▶); data reduction: *SAINT*; program(s) used to solve structure: *SHELXS97* (Sheldrick, 2008 ▶); program(s) used to refine structure: *SHELXL97* (Sheldrick, 2008 ▶); molecular graphics: *SHELXTL* (Sheldrick, 2008 ▶); software used to prepare material for publication: *SHELXL97*.

## Supplementary Material

Crystal structure: contains datablocks I, global. DOI: 10.1107/S1600536809034576/lh2890sup1.cif

Structure factors: contains datablocks I. DOI: 10.1107/S1600536809034576/lh2890Isup2.hkl

Additional supplementary materials:  crystallographic information; 3D view; checkCIF report

## Figures and Tables

**Table 1:** Hydrogen-bond geometry (Å, °)

*D*—H⋯*A*	*D*—H	H⋯*A*	*D*⋯*A*	*D*—H⋯*A*
N2—H2*A*⋯O1*W*^i^	0.86	1.90	2.713 (4)	156
N3—H3*A*⋯O3^ii^	0.86	1.99	2.825 (4)	162
O1*W*—H1*WB*⋯N4	0.84	2.02	2.852 (4)	177
O1*W*—H1*WA*⋯O2	0.84	2.25	3.018 (5)	154

Symmetry codes: (i) 
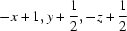
; (ii) 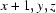.

**Fig. 1.**
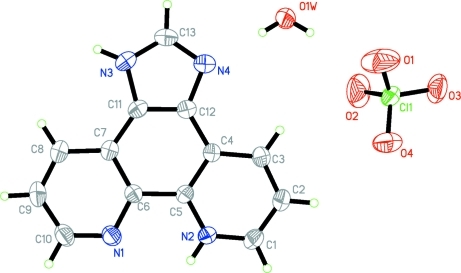
The asymmetric unit of (I) with displacement ellipdoids drawn at the the 30% probability level. H atoms are shown as spheres of arbitrary radii.

**Fig. 2.**
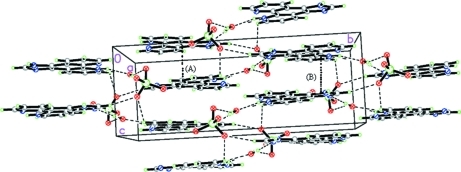
Part of the crystal structure of (I). Hydrogen bonds are drawn as dashed lines and π–π stacking interactions are denoted by dashed lines along with labels (A) and (B).

## supplementary crystallographic information

### Comment

1H-imidazo[4,5-f][1,10]-phenanthroline (IP) is an important derivative of 1,10-phenanthroline that has been used to recognize the secondary structure of DNA in an Ru(II) complex (Xiong *et al.*,  1999). IP is a good molecular building block and has been used to construct some interesting structures (Yu *et al.*, 2009, Liu *et al.*,  2009). In an attempt to form a Zn(II) complex with IP, we adventitiously formed the title compound (I) and its crystal structure is determined herein.

The asymmetric unit of (I) is shown in Fig 1. In the crystal structure N-H···O, O-H···N and O-H···O hydrogen bonds link cations, water molecules and perchlorate anions into a 2-D network (Fig. 2). Details of the hydrogen-bonding geometry are given in Table 1. In addition, there are weak π–π stacking interactions between layers, involving cations with centroid to centroid distances in the range 3.584 (2)-3.662 (2)Å forming a three-dimensional network.

### Experimental

IP (0.23 mg,0.1 mmol), Zn(ClO_4_)_2_ (0.27 mg, 0.1 mmol), were dissolved in  methanol. The mixture was heated and stirred for ten hours under reflux. The resulting solid was then filtered off to give a pure solution which was treated with diethyl ether in a closed vessel. Five weeks later, single crystals were obtained.

### Refinement

All H atoms were visible in difference Fourier maps but were subsequently placed in calculated positions treated as riding with C—H = 0.93, N—H == 0.86Å and with U_iso_(H) = 1.2U_eq_(C,N). The H atoms of the water molecules were included in the subsequent refinement with O-H = 0.84Å and U_iso_(H) = 1.5U_eq_(O).

### Crystal data

**Table:**

C_13_H_9_N_4_^+^·ClO_4_^−^·H_2_O	*F*(000) = 696
*M**_r_* = 338.71	*D*_x_ = 1.590 Mg m^−^^3^
Monoclinic, *P*2_1_/*c*	Mo *K*α radiation, λ = 0.71073 Å
Hall symbol: -P 2ybc	Cell parameters from 2521 reflections
*a* = 11.401 (2) Å	θ = 1.8–25.2°
*b* = 18.475 (3) Å	µ = 0.30 mm^−^^1^
*c* = 6.7163 (13) Å	*T* = 298 K
β = 90.179 (3)°	Block, colorless
*V* = 1414.7 (4) Å^3^	0.30 × 0.26 × 0.17 mm
*Z* = 4	

### Data collection

**Table:**

Bruker APEXII area-detector diffractometer	2534 independent reflections
Radiation source: fine-focus sealed tube	1734 reflections with *I* > 2σ(*I*)
graphite	*R*_int_ = 0.031
φ and ω scans	θ_max_ = 25.2°, θ_min_ = 1.8°
Absorption correction: multi-scan (*SADABS*; Bruker, 2005)	*h* = −13→11
*T*_min_ = 0.914, *T*_max_ = 0.950	*k* = −21→22
7051 measured reflections	*l* = −7→8

### Refinement

**Table:**

Refinement on *F*^2^	Primary atom site location: structure-invariant direct methods
Least-squares matrix: full	Secondary atom site location: difference Fourier map
*R*[*F*^2^ > 2σ(*F*^2^)] = 0.057	Hydrogen site location: inferred from neighbouring sites
*wR*(*F*^2^) = 0.197	H-atom parameters constrained
*S* = 1.01	*w* = 1/[σ^2^(*F*_o_^2^) + (0.1261*P*)^2^ + 0.1912*P*] where *P* = (*F*_o_^2^ + 2*F*_c_^2^)/3
2534 reflections	(Δ/σ)_max_ < 0.001
208 parameters	Δρ_max_ = 0.41 e Å^−^^3^
3 restraints	Δρ_min_ = −0.39 e Å^−^^3^

### Special details

**Table:**

Geometry. All esds (except the esd in the dihedral angle between two l.s. planes) are estimated using the full covariance matrix. The cell esds are taken into account individually in the estimation of esds in distances, angles and torsion angles; correlations between esds in cell parameters are only used when they are defined by crystal symmetry. An approximate (isotropic) treatment of cell esds is used for estimating esds involving l.s. planes.
Refinement. Refinement of F^2^ against ALL reflections. The weighted R-factor wR and goodness of fit S are based on F^2^, conventional R-factors R are based on F, with F set to zero for negative F^2^. The threshold expression of F^2^ > 2sigma(F^2^) is used only for calculating R-factors(gt) etc. and is not relevant to the choice of reflections for refinement. R-factors based on F^2^ are statistically about twice as large as those based on F, and R- factors based on ALL data will be even larger.

### Fractional atomic coordinates and isotropic or equivalent isotropic displacement parameters (Å^2^)

**Table:**

	*x*	*y*	*z*	*U*_iso_*/*U*_eq_	
N1	0.7565 (2)	0.41426 (15)	0.3687 (4)	0.0563 (7)	
N2	0.5235 (2)	0.38399 (13)	0.3439 (4)	0.0494 (7)	
H2A	0.5510	0.4273	0.3390	0.059*	
N3	0.8115 (3)	0.14741 (15)	0.3782 (4)	0.0539 (7)	
H3A	0.8855	0.1388	0.3832	0.065*	
N4	0.6190 (3)	0.12600 (14)	0.3669 (4)	0.0537 (7)	
C1	0.4079 (3)	0.37511 (19)	0.3383 (5)	0.0583 (9)	
H1	0.3590	0.4153	0.3301	0.070*	
C2	0.3602 (3)	0.30690 (19)	0.3445 (5)	0.0577 (9)	
H2	0.2792	0.3008	0.3435	0.069*	
C3	0.4328 (3)	0.24803 (18)	0.3521 (4)	0.0508 (8)	
H3	0.4011	0.2016	0.3527	0.061*	
C4	0.5552 (2)	0.25749 (16)	0.3589 (4)	0.0423 (7)	
C5	0.5997 (2)	0.32858 (16)	0.3569 (4)	0.0424 (7)	
C6	0.7246 (3)	0.34396 (16)	0.3670 (4)	0.0437 (7)	
C7	0.8053 (3)	0.28627 (17)	0.3736 (4)	0.0463 (7)	
C8	0.9257 (3)	0.3041 (2)	0.3816 (5)	0.0575 (9)	
H8	0.9826	0.2681	0.3859	0.069*	
C9	0.9563 (3)	0.3750 (2)	0.3827 (5)	0.0676 (10)	
H9	1.0350	0.3880	0.3883	0.081*	
C10	0.8704 (3)	0.4282 (2)	0.3753 (5)	0.0656 (10)	
H10	0.8942	0.4763	0.3751	0.079*	
C11	0.7577 (3)	0.21528 (16)	0.3725 (4)	0.0462 (7)	
C12	0.6393 (3)	0.20018 (16)	0.3648 (4)	0.0451 (7)	
C13	0.7259 (3)	0.09872 (19)	0.3745 (5)	0.0584 (9)	
H13	0.7402	0.0492	0.3770	0.070*	
Cl1	0.15290 (7)	0.11627 (5)	0.40411 (14)	0.0643 (4)	
O1	0.2077 (4)	0.07296 (19)	0.5458 (6)	0.1414 (17)	
O2	0.2199 (3)	0.1137 (2)	0.2286 (6)	0.1356 (15)	
O3	0.0402 (2)	0.08894 (19)	0.3635 (5)	0.0990 (10)	
O4	0.1451 (3)	0.18729 (16)	0.4828 (6)	0.1099 (12)	
O1W	0.4428 (2)	0.02582 (13)	0.2434 (5)	0.0871 (9)	
H1WB	0.4934	0.0564	0.2771	0.131*	
H1WA	0.3755	0.0409	0.2713	0.131*	

### Atomic displacement parameters (Å^2^)

**Table:**

	*U*^11^	*U*^22^	*U*^33^	*U*^12^	*U*^13^	*U*^23^
N1	0.0558 (17)	0.0523 (17)	0.0608 (16)	−0.0063 (13)	−0.0055 (13)	0.0023 (12)
N2	0.0472 (15)	0.0436 (15)	0.0572 (16)	0.0054 (11)	−0.0031 (12)	0.0011 (11)
N3	0.0536 (16)	0.0575 (17)	0.0506 (15)	0.0156 (14)	0.0007 (12)	0.0021 (12)
N4	0.0655 (18)	0.0469 (16)	0.0488 (15)	0.0036 (13)	0.0019 (13)	0.0006 (11)
C1	0.050 (2)	0.064 (2)	0.061 (2)	0.0124 (16)	−0.0055 (15)	−0.0016 (15)
C2	0.0421 (17)	0.069 (2)	0.062 (2)	0.0021 (16)	−0.0014 (15)	−0.0016 (16)
C3	0.0482 (18)	0.057 (2)	0.0470 (17)	−0.0054 (15)	−0.0012 (13)	0.0004 (14)
C4	0.0434 (17)	0.0471 (17)	0.0364 (15)	−0.0003 (13)	0.0031 (12)	−0.0003 (12)
C5	0.0421 (16)	0.0479 (17)	0.0372 (15)	0.0035 (13)	−0.0024 (12)	0.0004 (12)
C6	0.0469 (17)	0.0445 (17)	0.0396 (15)	−0.0030 (13)	−0.0012 (12)	0.0032 (11)
C7	0.0434 (16)	0.057 (2)	0.0381 (15)	0.0013 (14)	0.0004 (12)	0.0020 (12)
C8	0.0431 (18)	0.071 (2)	0.0581 (19)	0.0031 (16)	−0.0005 (14)	0.0023 (16)
C9	0.044 (2)	0.086 (3)	0.074 (2)	−0.0140 (18)	−0.0041 (17)	0.0049 (19)
C10	0.062 (2)	0.059 (2)	0.076 (2)	−0.0161 (18)	−0.0063 (18)	0.0054 (17)
C11	0.0507 (18)	0.0493 (19)	0.0386 (15)	0.0098 (14)	0.0016 (13)	0.0023 (12)
C12	0.0511 (18)	0.0476 (18)	0.0366 (15)	0.0031 (14)	0.0033 (12)	0.0001 (12)
C13	0.078 (2)	0.0406 (18)	0.0570 (19)	0.0052 (18)	−0.0009 (17)	−0.0016 (14)
Cl1	0.0453 (5)	0.0575 (6)	0.0902 (7)	0.0079 (4)	−0.0048 (4)	−0.0043 (4)
O1	0.157 (4)	0.089 (2)	0.178 (4)	0.033 (2)	−0.096 (3)	0.005 (2)
O2	0.100 (3)	0.167 (4)	0.140 (3)	−0.013 (2)	0.059 (2)	−0.016 (3)
O3	0.0470 (15)	0.117 (2)	0.133 (3)	−0.0055 (15)	−0.0056 (16)	−0.028 (2)
O4	0.107 (3)	0.0571 (18)	0.166 (3)	0.0161 (16)	−0.005 (2)	−0.0134 (19)
O1W	0.0592 (16)	0.0560 (16)	0.146 (3)	−0.0021 (12)	0.0052 (16)	−0.0208 (15)

### Geometric parameters (Å, °)

**Table:**

N1—C10	1.324 (4)	C5—C6	1.454 (4)
N1—C6	1.349 (4)	C6—C7	1.408 (4)
N2—C1	1.329 (4)	C7—C8	1.413 (4)
N2—C5	1.345 (4)	C7—C11	1.419 (4)
N2—H2A	0.8600	C8—C9	1.355 (5)
N3—C13	1.327 (4)	C8—H8	0.9300
N3—C11	1.396 (4)	C9—C10	1.388 (5)
N3—H3A	0.8600	C9—H9	0.9300
N4—C13	1.320 (4)	C10—H10	0.9300
N4—C12	1.390 (4)	C11—C12	1.379 (4)
C1—C2	1.373 (5)	C13—H13	0.9300
C1—H1	0.9300	Cl1—O1	1.390 (3)
C2—C3	1.367 (5)	Cl1—O3	1.406 (3)
C2—H2	0.9300	Cl1—O2	1.407 (4)
C3—C4	1.408 (4)	Cl1—O4	1.417 (3)
C3—H3	0.9300	O1W—H1WB	0.8379
C4—C5	1.408 (4)	O1W—H1WA	0.8377
C4—C12	1.429 (4)		
			
C10—N1—C6	116.9 (3)	C6—C7—C11	116.7 (3)
C1—N2—C5	123.2 (3)	C8—C7—C11	126.0 (3)
C1—N2—H2A	118.4	C9—C8—C7	118.4 (3)
C5—N2—H2A	118.4	C9—C8—H8	120.8
C13—N3—C11	106.6 (3)	C7—C8—H8	120.8
C13—N3—H3A	126.7	C8—C9—C10	120.2 (3)
C11—N3—H3A	126.7	C8—C9—H9	119.9
C13—N4—C12	102.9 (3)	C10—C9—H9	119.9
N2—C1—C2	120.3 (3)	N1—C10—C9	123.7 (3)
N2—C1—H1	119.8	N1—C10—H10	118.1
C2—C1—H1	119.8	C9—C10—H10	118.1
C3—C2—C1	119.4 (3)	C12—C11—N3	104.4 (3)
C3—C2—H2	120.3	C12—C11—C7	124.2 (3)
C1—C2—H2	120.3	N3—C11—C7	131.4 (3)
C2—C3—C4	120.2 (3)	C11—C12—N4	111.2 (3)
C2—C3—H3	119.9	C11—C12—C4	120.5 (3)
C4—C3—H3	119.9	N4—C12—C4	128.3 (3)
C3—C4—C5	118.2 (3)	N4—C13—N3	114.9 (3)
C3—C4—C12	125.0 (3)	N4—C13—H13	122.6
C5—C4—C12	116.8 (3)	N3—C13—H13	122.6
N2—C5—C4	118.6 (3)	O1—Cl1—O3	109.5 (2)
N2—C5—C6	119.1 (3)	O1—Cl1—O2	108.1 (3)
C4—C5—C6	122.3 (3)	O3—Cl1—O2	108.9 (2)
N1—C6—C7	123.5 (3)	O1—Cl1—O4	107.8 (2)
N1—C6—C5	116.9 (3)	O3—Cl1—O4	110.2 (2)
C7—C6—C5	119.5 (3)	O2—Cl1—O4	112.2 (2)
C6—C7—C8	117.3 (3)	H1WB—O1W—H1WA	110.3

### Hydrogen-bond geometry (Å, °)

**Table:**

*D*—H···*A*	*D*—H	H···*A*	*D*···*A*	*D*—H···*A*
N2—H2A···O1W^i^	0.86	1.90	2.713 (4)	156
N3—H3A···O3^ii^	0.86	1.99	2.825 (4)	162
O1W—H1WB···N4	0.84	2.02	2.852 (4)	177
O1W—H1WA···O2	0.84	2.25	3.018 (5)	154

Symmetry codes: (i) −*x*+1, *y*+1/2, −*z*+1/2; (ii) *x*+1, *y*, *z*.
